# Visual Field Sensitivity Prediction Using Optical Coherence Tomography Analysis in Hydroxychloroquine Toxicity

**DOI:** 10.1167/iovs.63.1.15

**Published:** 2022-01-11

**Authors:** Gopal Jayakar, Tharindu De Silva, Catherine A. Cukras

**Affiliations:** 1 Unit on Clinical Investigation of Retinal Disease, Division of Epidemiology & Clinical Applications, National Eye Institute, National Institutes of Health, Bethesda, Maryland, United States

**Keywords:** hydroxychloroquine, machine learning, visual field, optical coherence tomography, retina

## Abstract

**Purpose:**

This study investigates the association between local retina structure and visual function in a cohort with long-term hydroxychloroquine (HCQ) use.

**Methods:**

The study included 84 participants (54 participants without toxicity and 30 participants with toxicity) with history of chronic HCQ use (14.5 ± 7.4 years) who had testing with spectral-domain optical coherence tomography (SD-OCT) imaging and Humphrey 10-2 visual fields. Optical coherence tomography (OCT) metrics (total and outer retina thickness [TRT and ORT], minimum intensity [MinI], and ellipsoid zone [EZ] loss) were sampled in regions corresponding to visual field test locations. Univariate linear correlations were investigated and a multivariate random forest regression using a combination of OCT metrics was used to predict visual field sensitivity by locus using a leave-one-out cross-validation strategy.

**Results:**

In univariate linear regression, EZ loss demonstrated the strongest relationship with visual field sensitivities in the parafoveal ring with R^2^ = 0.58. TRT and ORT revealed positive correlations with visual field sensitivity (R^2^ = 0.57 and 0.40, respectively), whereas total and outer retinal MinI yielded negative correlations (R^2^ = 0.10 and 0.22). The multivariate model improved correlations (R^2^ = 0.66) yielding a root mean squared error of 3.8 decibel (dB). Feature importance analysis identified EZ loss as the most relevant predictor of function.

**Conclusions:**

Multiple OCT-derived quantitative metrics used in combination can provide information to predict local sensitivities. The results indicate a strong relationship between retinal function and OCT measures, which contribute to the understanding of the retinal toxicity caused by HCQ as well as being applicable to outcome development for other degenerative diseases of the outer retina.

Hydroxychloroquine (HCQ) is prescribed to treat and prevent malaria and is used chronically as a first-line drug to treat lupus, arthritis, and other autoimmune conditions. Among long-term users, possible side effects include visual dysfunction resulting from retinal damage.^1^ The American Academy of Ophthalmology (AAO) issues recommendations for screening patients using HCQ which include at its core, annual screening tests including subjective functional evaluations with perimetry and objective evaluations of structure using spectral domain optical coherence tomography (SD-OCT).^1^ Although the goal of screening is to identify any evidence of visual dysfunction, obtaining objective structural evidence to corroborate visual field findings before diagnosing toxicity is recommended as spurious changes in the visual field can be triggered by nonpathological causes or nonretinal causes.^1^ Clinical evaluations with congruent findings on two modalities increase the probability of identifying true instances of pathology.

Clinical evaluation of optical coherence tomography (OCT) images have relied on qualitative inspections of OCT features, with attention to EZ attenuation and loss.^2^^,^^3^ Some reports have commented on OCT changes that precede visual field changes,^4^ whereas others have reported on groups that demonstrate changes in visual field prior to appreciation of structural changes.^5^ Further investigations have demonstrated that quantitative analysis of OCT can reveal changes correlated with toxicity.^6^^–^^8^

A model to assess point-to-point correlations between perimetry derived sensitivities and structural metrics obtained from SD-OCT would be desirable to validate changes on each modality that may be overlooked in isolation but may be corroborative when evaluated in tandem. Strong structure-function relationships thus established could also be helpful in some settings where functional tests are infeasible.

In this study, we utilize data from a study of patients on long-term HCQ use to study correlations between humphrey visual field (HVF) 10-2 and SD-OCT testing performed at the same visit. The study subjects consist of a cohort of participants with no evidence of HCQ toxicity and a cohort with a range of HCQ toxicity severities. Point-to-point correlations are investigated both individually with each OCT-based measure and in combination after building a machine learning model that predicts the functional sensitivity in an area of retina using several structural metrics including: ellipsoid zone loss (EZ loss), retina thickness (RT), minimum intensity (MinI), and mean intensity (MeanI). Exploration of structure-function correlation facilitates the identification of structural features that are indicative of functional deficiencies.

## Methods

### Participants

Data were collected as part of the institutional review board approved prospective study at the National Eye Institute, Bethesda, MD (www.clinicaltrials.gov identifier NCT01145196). The study adhered to the tenets of the Declaration of Helsinki and the Health Insurance Portability and Accountability Act and signed informed consent was obtained from all participants. All participants were long term (>5 years) HCQ users and did not have any history of other concomitant retinal disease. A subset of the patient cohort has been the subject of previous analyses.^6^^,^^8^

### Study Procedures

Each participant underwent an ocular examination, including automated 10-2 Humphrey perimetry, and SD-OCT imaging. Testing was performed in both eyes of each participant. Perimetric assessment was performed using a standard 10-2 Humphrey Visual Field Analyzer (Humphrey Instruments, Inc., San Leandro, CA, USA) with a white test spot. The visual field mean deviation (VFMD) values and pattern standard deviation (PSD) were obtained from the visual field output. The raw 10-2 visual field sensitivity data were extracted at each of the 68 loci for each of the sampled eyes. Multifocal electroretinography (mfERG) testing was also performed on all participants according to the International Society for Clinical Electrophysiology of Vision guidelines,^9^ based on the 61-hexagon stimulus pattern of the VERIS Clinic system (Electro-Diagnostic Imaging, Inc., Redwood, CA, USA). The average response densities (nanovolts per degrees squared) within concentric rings from the center (ring 1) to the periphery (ring 5) were generated by the mfERG VERIS software. The ring ratios of the mfERG were defined as ratios of the central hexagon amplitude (R1) to each of the peripheral ring amplitudes (R2–R5). These ratios were calculated for all tested eyes.

The SD-OCT images were acquired using the Heidelberg Spectralis HRA-OCT system (Spectralis; Heidelberg Engineering, Heidelberg, Germany). Macular cube scans were comprised of 121 horizontal B-scans with 60 µm spacing and spanned 30 degrees horizontally and 25 degrees vertically.

### Toxicity Determination

The determination of toxicity was based on the AAO's 2016 recommendations and included the primary recommended screening tests of automated visual field testing and SD OCT.^1^ Our dataset also included mfERG testing, an objective test of retina function, to corroborate visual field testing. The presence of at least three contiguous abnormal points on the pattern deviation map (*P* < 2%) or a full ring scotoma was used as criteria for determining abnormalities in the visual field.^10^ The presence of either of the following two conditions: (1) increased R1-to-R2 ratio (defined as exceeding the 99% confidence limits for the normal population), or (2) reduced R1 absolute amplitude (defined as less than the 99% confidence limits for the normal population) on mfERG testing was used as evidence of toxicity.^8^^,^^11^ These criteria are summarized in Supplementary Table S1.

For some analyses and illustrations, severity groupings based upon the extent of photoreceptor loss observed in the foveal B scan were utilized.^6^ Mild severity (group 1) is typified by little or no appreciable EZ loss on the foveal B scan (<=100 µm), group 2 having mild-moderate EZ loss (100 µm < EZ loss <=1000 µm), group 3 having severe loss preserving the foveal island (>500 µm) but with EZ loss >1000 µm, and group 4 with severe loss and foveal involvement (Supplementary Fig. S1).

### Data Operations

Measurements related to EZ loss, RT, MinI, and MeanI were derived from SD-OCT images. Areas of EZ loss were identified using an automatic deep-learning based algorithm with manual verification of contours reviewed by a retina specialist (authorC.C.). The algorithm had been compared to manual ground truth contouring of EZ loss and demonstrated excellent (0.98) correlation with those areas.^12^^,^^13^ The algorithm first operated on individual B-scans and then produced an enface binary map depicting EZ loss regions.

To compute thickness and intensity-based measurements, SD-OCT volume was automatically segmented using OCT-Explorer (version 3.8; University of Iowa, Iowa City, IA, USA).^14^^–^^16^ Three segmentation contours were derived and manually verified: (1) inner limiting membrane (ILM), (2) outer plexiform layer (OPL; outer boundary of the inner nuclear layer [INL]), and (3) retinal pigment epithelium (RPE) (outer boundary). These contours were manually inspected for accuracy and adjusted (*n* = 7 eyes) when necessary by a trained expert and verified by a retina specialist (author C.C.). Thickness measurements were directly computed using the segmented contours for total retina thickness (TRT) as the distance from the inner limiting membrane to the retinal pigment epithelium and outer retinal thickness (ORT) as the distance from the OPL to the RPE.

Prior to computing intensity-based measurements, image intensities were normalized by performing two image preprocessing steps. First, an edge-preserved smoothing operation was applied using a bilateral filter to decrease noise^17^ and mitigate the effect of speckle-like local intensity fluctuations in OCT. Second, the image intensities were multiplied by a scaling factor such that the average intensity within the inner retina was constant across the images. This normalization step accounted for intensity level differences across B-scans due to signal differences between acquisitions. After the normalization step, the mean pixel intensity of the inner retina is approximately the same across all eyes. The mean and minimum measurements were then calculated along each A-scan within the coordinates of the segmented contours. The intensity metrics computed were MeanI and MinI, assessed across the outer retina (OR MeanI, OR MinI) and total retina (TR MeanI, TR MinI).

**Figure 1.**
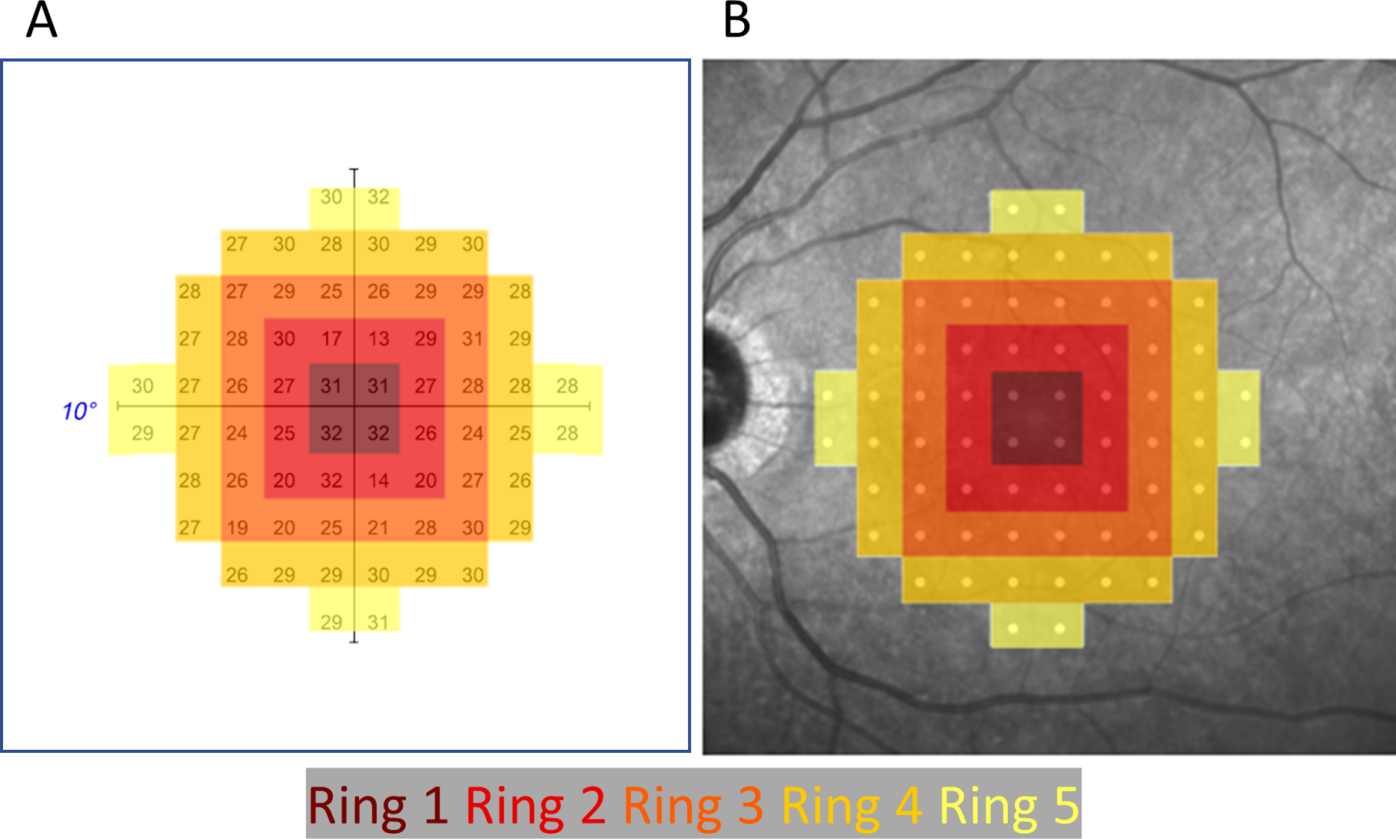
Ring designations applied to each of the HVF loci. Concentric regions are identified with ring numbers, starting with ring 1 at the central points through ring 5 at the outside extremities of the VF.

A digital scaffold was created to match the test locations in the 10-2 HVF and consisted of a grid of 68 circular spots with a 288 µm diameter spaced 576 µm (2 degrees) apart. The scaffold was automatically registered to the fovea on the OCT en face scans (Fig. 1) and OCT measures (thickness measures, intensity measures, and EZ loss) in the scaffold locations were calculated. Contours from OCT layer segmentation were used to calculate thickness measures in the test locations. Intensity values were obtained by averaging the intensity in the test location area using a test spot diameter that was increased to 576 µm (2 degrees) to provide a more robust metric for minimum intensity. EZ loss in the test location was represented as a fraction of the test area with values ranging from 0 (no EZ loss within the test locus) to 1 (EZ loss over the entire test locus).

### Correlation Analysis – Univariate Model

For each OCT metric (EZ Loss, TRT, ORT, TR MeanI, OR MeanI, TR MinI, and OR MinI), univariate relationships with visual field sensitivity at the corresponding locus were calculated using the python NumPy version 1.18.5 linear regression implementation.^18^ To control for effects related to the position of the test spot relative to the fovea, some analyses made use of a ring analysis as shown in Figure 1B. Ring 1 (dark red) comprised the 4 central test points, and ring 2 (red) included the parafoveal points. When performing correlations across all test spots, normalization was performed by computing the Z-score at each locus to allow better comparison of loci at different locations within the eye^19^ according to $Z-\mathrm{score}=\frac{x-\mu_{unaffected}}{\sigma_{unaffected}}$, where *x* is the value of a sample at a locus, µ and σ are the mean and standard deviation, respectively, for the unaffected samples at the corresponding locus. After grouping test points in each ring, univariate linear analysis was performed to assess correlation with OCT metrics.

### Correlation Analysis – Multivariate Model

A random forest (RF) machine learning model was used to predict visual field sensitivity using a combination of OCT derived metrics. The RF model was selected due to its ability to find nonlinear relationships in a multidimensional space without requiring large data sets (compared to deep-learning approaches). It also enables the evaluation of contributing factors via feature importance analysis.^20^ The model was implemented after including nine features as input: EZ Loss, TRT, ORT, TR MeanI, OR MeanI, TR MinI, OR MinI, x-coordinate, and y-coordinate relative to the fovea. Due to the small number of input features (*n* = 9), an explicit feature reduction method was not implemented. These metrics were chosen due to established correlations with visual function. The model was trained to predict the visual field sensitivity at the corresponding locus with mean-squared-error loss criterion. Multiple models were trained in a leave-one-patient-out (LOPO) cross-validation approach where both eyes of the patients were left out when training a single model. Training was performed with bootstrapping where randomly selected data points were used to construct decisions trees to reduce overfitting. Five hundred random estimators^20^ and 10,474 maximum samples during bootstrapping were selected empirically as parameters of the model during training. For each eye, predictions were made locus by locus, and were then compared to the ground truth visual field values. Root mean square error (RMSE) and mean absolute error (MAE) were calculated across all predictions for all eyes. Feature importance of the models were analyzed to understand the contribution of different metrics in predicting visual field sensitivity.

## Results

Eighty-four participants with a history of HCQ usage lasting more than 5 years (mean = 14.5 ± 7.4 years) had HVF 10-2 testing, mfERG testing, and SD-OCT images available in each eye for analysis. Using objective criteria applied to the automated visual field testing, OCT and mfERG (see Supplementary Table S1), 54 individuals were found to have no evidence of toxicity, and 30 had evidence of toxicity. Four patients with toxicity had one eye that met the criteria for toxicity and fellow eyes that demonstrated abnormal mfERG ring ratios but without the greater than three contiguous abnormal points on the pattern deviation plot (*P* < 2%). These eyes were classified as having toxicity (mild). One eye from an affected patient was excluded due to misaligned OCT B-scans.

The 59 eyes with toxicity had a range of severities: 16 eyes from 10 participants had mild changes and were group 1, 8 eyes from 5 participants were classified as group 2, 24 eyes from 15 participants were classified as group 3, and 11 eyes from 7 participants had severe changes and were classified as group 4. The demographics of the groups are listed in Table 1 with patients having eyes with different severity scores described by the more severe grade. There were statistically significant differences in age between the unaffected group and group 3 as well as the unaffected group and group 4. Beyond that, no two groups exhibited significant differences in age, duration of HCQ dosage, or the average dosage to body weight ratio (not shown in the table).

**Table 1.** Description of the Patient Cohorts Included in the Analysis

Group	Number of Patients	Age ± Standard Deviation	% Female	Avg HCQ Duration (Years) ± Standard Deviation	% > 5 mg/kg
Unaffected	54	56.5 ± 11.6	92.6	14.5 ± 7.4	62.96
1	7	60.7 ± 15	85.7	16.7 ± 8.7	66.67
2	4	54 ± 14.6	100	12.2 ± 3.4	50
3	12	64.7 ± 8.6*	91.7	12 ± 5.2	80
4	7	69.6 ± 4.7*	100	16.7 ± 7.1	66.67

Asterisks denote statistically significant differences from the unaffected population. No statistically significant differences were observed among groups 1 to 4 for any metric. Patients are grouped in the more severe group between their two eyes.

**Figure 2.**
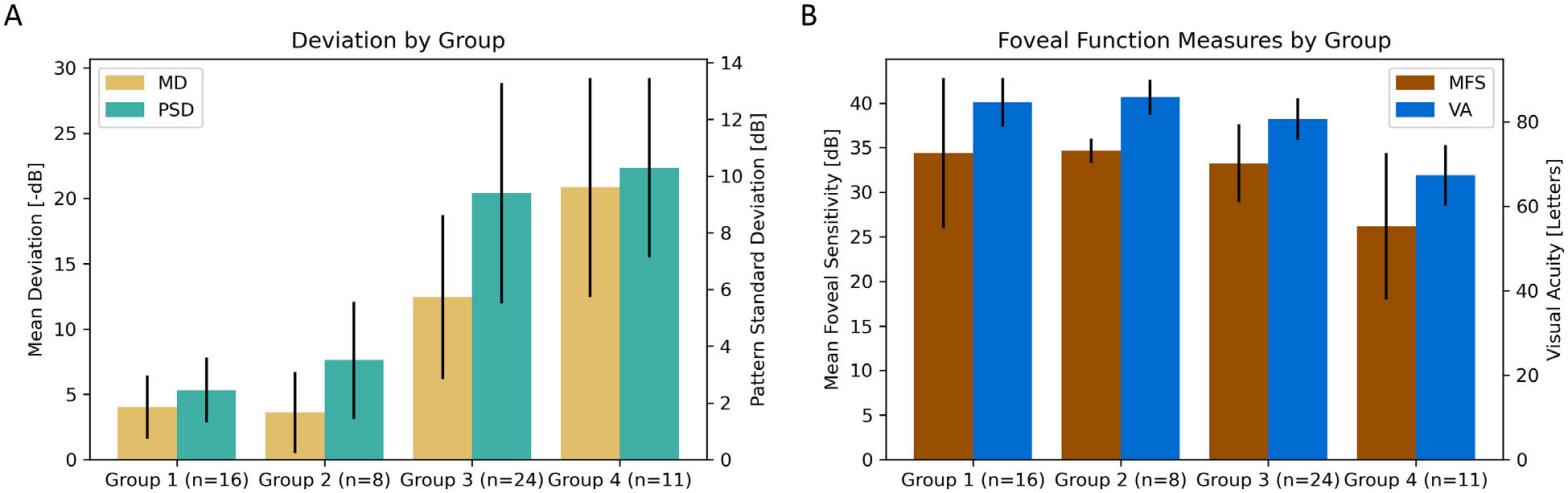
(**A**) Mean deviation (MD) and pattern standard deviation (PSD) by affected status and toxicity severity group. Error bars indicate one standard deviation. (**B**) Mean foveal sensitivity (MFA) and visual acuity (VA) by affected status and toxicity severity group.

Figure 2 demonstrates that in eyes with evidence of toxicity, the negative HVF MD worsens monotonically with increasing toxicity severity. The PSD increases with increasing severity group until group 3 beyond which PSD does not increase significantly. Visual acuity and mean foveal sensitivity of group 4 eyes was significantly worse than unaffected eyes and more mildly affected eyes. Because these group 4 eyes have poor acuity and fixation, they were excluded from local point-to-point structure function analyses as the visual field tests rely on foveal fixation to infer the location of the test points.

**Figure 3.**
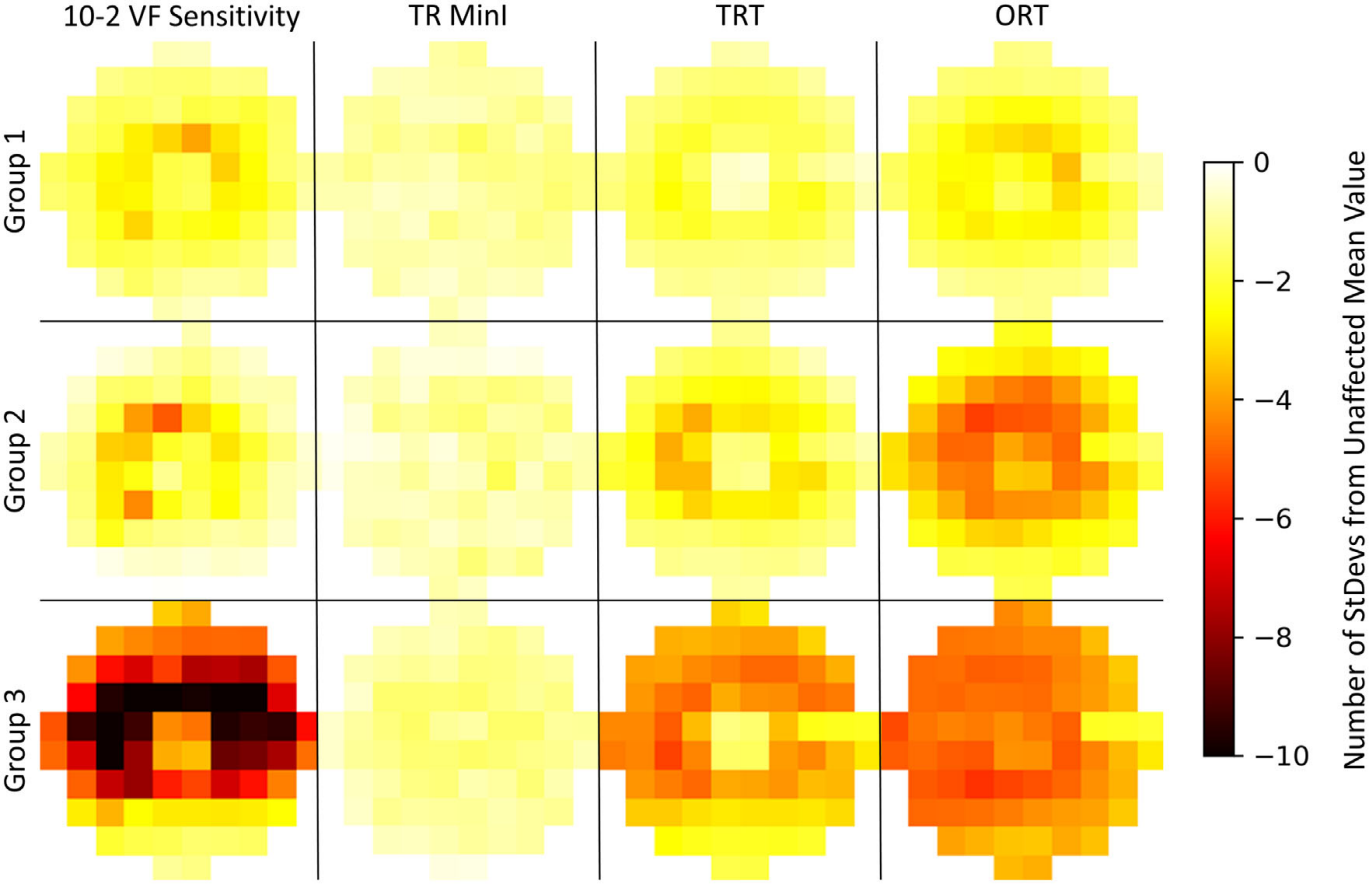
The modified z-scores for functional testing and structural assessments at each test point averaged over the participants in each affected severity group. The data are presented in VF view and as right eye data.

One hundred fifty-six eyes (108 eyes without toxicity and 48 eyes with evidence of toxicity) underwent structure-function pointwise analyses. Modified z scores for all points in affected eyes (*n* = 48) were calculated using the unaffected eyes as the reference. Data were aggregated across severity groups and then averaged at each point within each affected severity group. The averaged visual field maps in Figure 3 demonstrate a reduction of visual field sensitivities in a parafoveal configuration in group 1 eyes which becomes denser and more extensive with increasing group severity. The total retina thickness reflects similar changes with more significant decreases highlighted in the outer retina in groups 1 and 2. The minimum intensity also has deviation from the reference population in the parafoveal area that increases with increased severity but has a smaller range of abnormal Z scores than the other measures.

### Univariate Analysis

**Figure 4.**
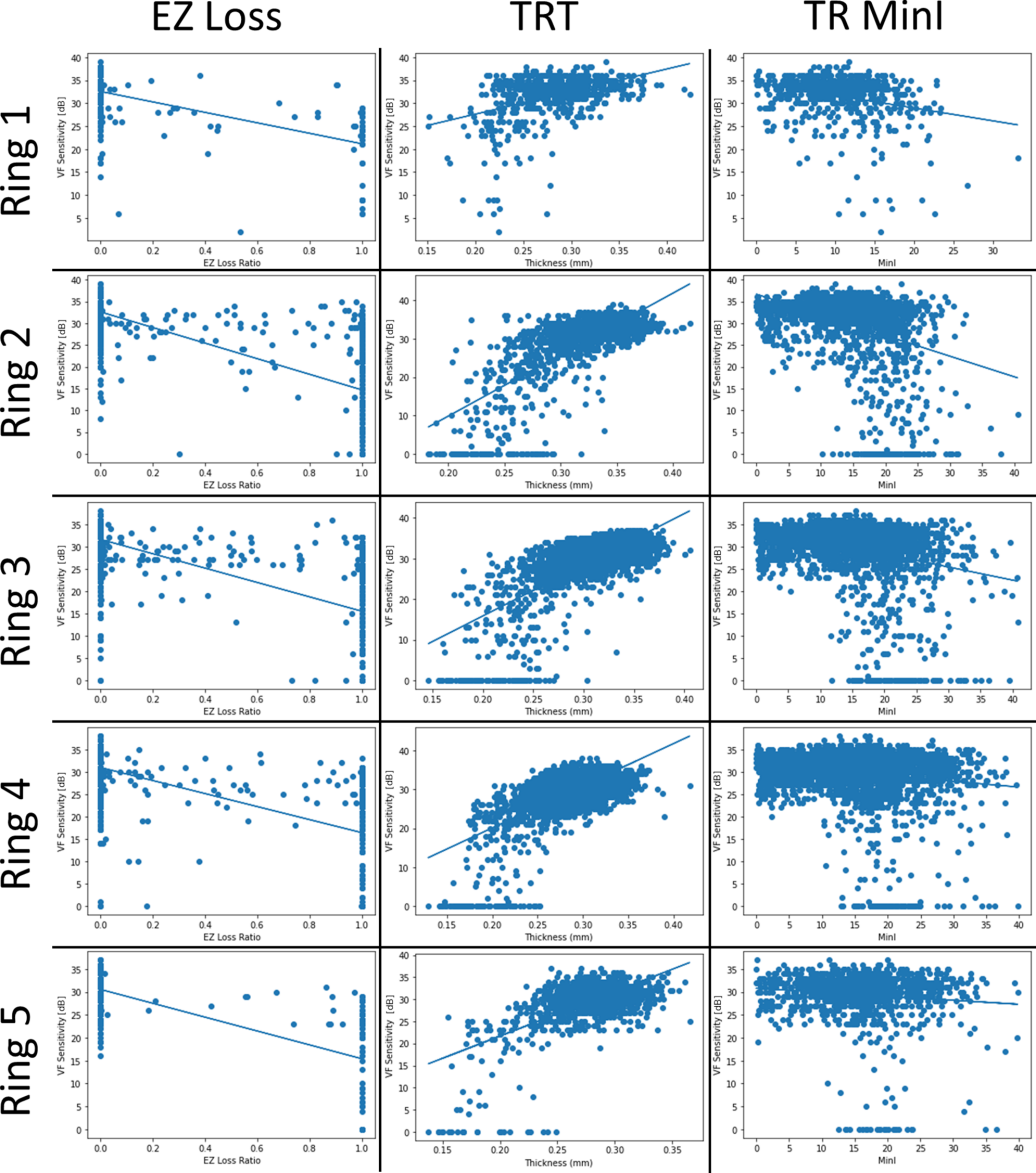
Correlations between EZ Loss (*left*), TRT (*middle*), and total retinal minimum intensity (*right*) with VF sensitivity across the different rings. Each locus in each eye is represented.

Univariate relationships between functional sensitivities and each OCT metric were explored with grouped analyses consisting of points equidistant (with small exception at the corners) from the fovea within each ring (Fig. 4, Supplementary Fig. S2). The strongest relationship with visual field sensitivity among all OCT metrics investigated was found with EZ loss in ring 2, the parafoveal ring, R^2^ = 0.58 with an MAE of 3.42 dB (standard deviation = 4.1 dB). The MinI within each scan region displayed the least correlation with function; TR and OR MinI yielded R^2^ = 0.10 and 0.22 in ring 2, respectively, and MAE = 5.2 dB (standard deviation = 5.7 dB) and 5.0 (standard deviation = 5.3 dB), respectively (see Supplementary Fig. S2). Total retinal thickness was positively associated with sensitivity throughout all rings. In rings 2, 3, and 4, R^2^ ≥ 0.43 with TRT MAE ≤ 3.77 dB. ORT and sensitivity in rings 2, 3, and 4 presented weaker correlations than TRT and sensitivity (see Supplementary Fig. S2). However, the ORT correlation within the fovea (R^2^ = 0.39) exceeded that of TRT (R^2^ = 0.18).

### Multivariate Analysis

Multivariate random forest models were constructed using multiple OCT metrics. For each patient, the random forest (RF) model was trained to predict the visual field sensitivity by locus using a leave-one-out cross validation approach, dropping both eyes from the patient in question. By comparing the performance of the predicted sensitivities to the measured visual sensitivities, our study calculated multiple measures of error assessments, as shown in Table 2. The MAE for an individual locus prediction across all eyes was 2.5 dB with a standard deviation of 2.87 dB for the absolute errors. The RMSE of the RF model was 3.84 dB for each locus prediction across all eyes (R^2^ = 0.66). Comparing the average of the sensitivity values calculated from the RF model output across all loci in an individual eye to the measured mean visual field sensitivity, the MAE of the mean sensitivity was found to be 1.69 dB.

**Table 2.** Summary of Metrics Reporting RF Model Performance

Type of Error	Mean	SD	95% CI
Absolute error, locus	2.5 dB	2.87 dB	2.49–2.60 dB
Real error, locus	−0.03 dB	3.84 dB (RMSE)	−7.56 to –7.5 dB

Mean error is the average signed error for all predictions of VF sensitivity at a locus. 95% confidence interval for error represents the 95% confidence interval for a single measurement, where the 95% confidence interval for mean error represents the same for the mean error in repeated trials.

**Figure 5.**
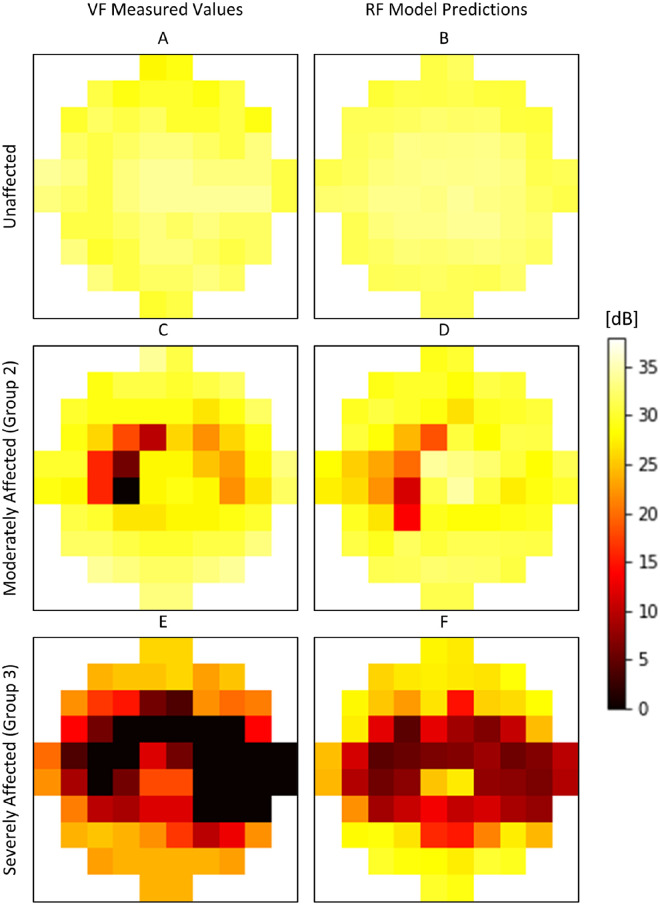
Comparison of measured VF sensitivities (*left*) and RF-predicted sensitivities (*right*) derived only from OCT metrics. Individual eyes from unaffected (**A, B**), group 2 toxicity (**C, D**), and group 3 toxicity (**E, F**) are shown.

Figure 5 illustrates examples of the model's performance for three eyes: an unaffected eye, an eye with moderate toxicity, and an eye with severe toxicity and demonstrates the similarity of sensitivity patterns in the prediction maps compared to the actual measured visual fields.

**Figure 6.**
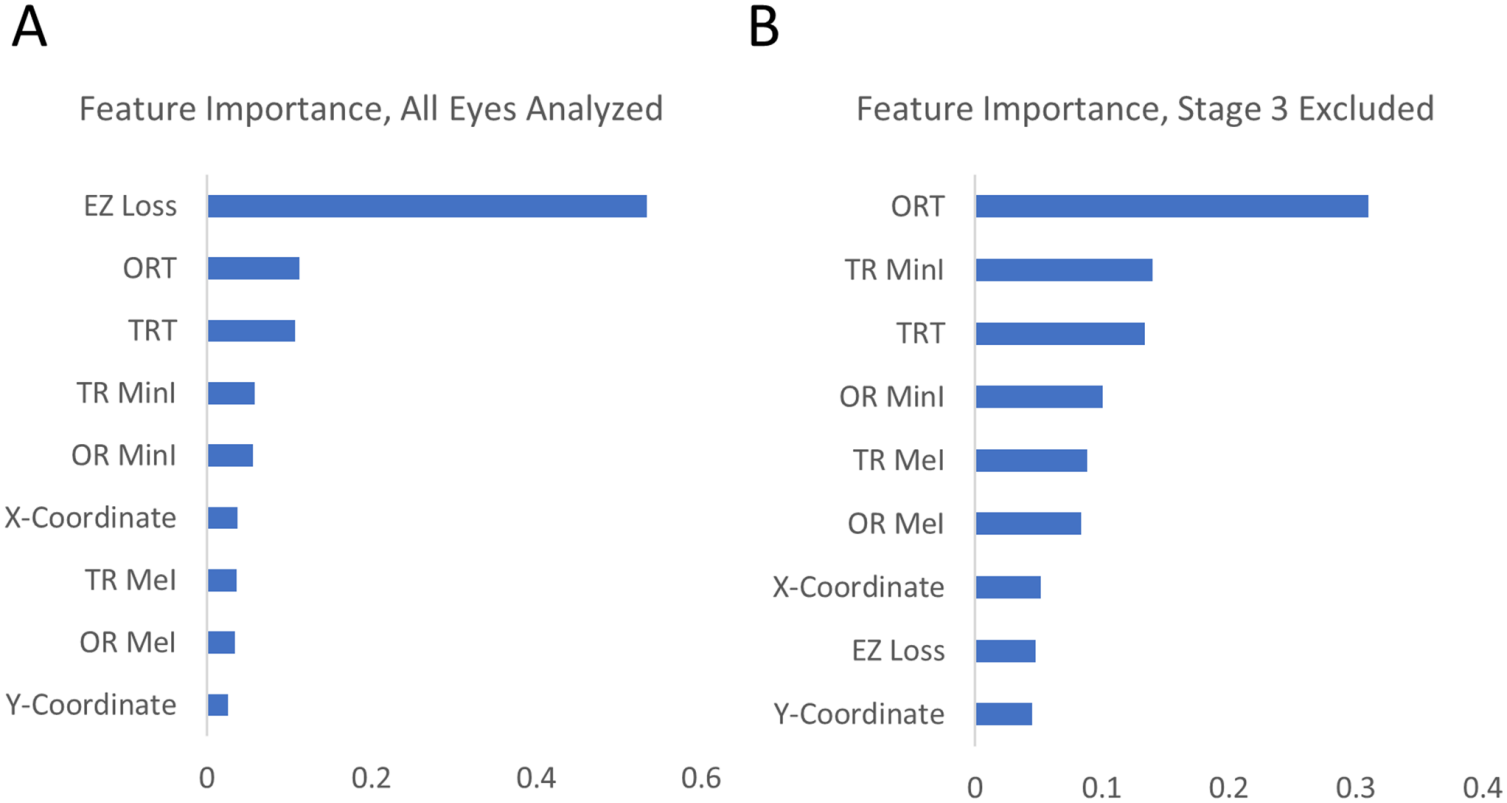
Average importance of each feature aggregated from the RF models trained for each patient. When trained on the entire dataset (**A**), the EZ loss feature showed greatest importance. EZ loss became least important in the models trained only with unaffected/mildly affected datasets (**B**).

The RF model can also be interrogated to identify the features (i.e. metrics) contributing the greatest to the model's prediction. Feature importance metrics for each of the nine features provided are found in Figure 6 and demonstrate that the most important feature in the model was EZ loss with importance of 0.53. TRT and ORT were the second and third most important, both with importance values of 0.11. These results conform with the high R^2^ values each of these displayed in the univariate linear correlations relative to the other metrics. To identify features important in eyes with little to no EZ loss, a separate RF model was trained on data limited to unaffected and mildly affected samples. With this model, the EZ loss feature displayed decreased importance, whereas ORT was found to be the most important feature in this subset. These metrics provide an understanding of which OCT measures were most weighted in the model's predictions.

## Discussion

In these point-to-point structure-function analyses in patients treated with HCQ, we identify multiple OCT-derived quantitative metrics that exhibit correlation with visual field sensitivity. Previous analyses of HCQ toxicity using the Early Treatment Diabetic Retinopathy Study (ETDRS) grid have identified OCT-derived metrics such as EZ loss, thickness measures such as TRT and ORT, and intensity-based measures such as MinI as being relevant and correlated to visual field mean deviations measured across the retina^6^^,^^21^ or to the visual field sensitivity in the ETDRS subfield.^21^ Our analyses build upon these results to resolve what aspects of structural change are driving retinal function in each tested location. Across univariate ring-grouped analysis correlations (see Fig. 4, Supplementary Fig. S2) as well as multivariate analyses using RF model, our study validates EZ loss and retina thickness (ORT and TRT) as features that hold the most relevance in predicting visual function from retina structure measured in OCT.

Although univariate analysis across the entire spectrum of disease analyzed demonstrates the best correlation with EZ loss, limiting the analysis to eyes with mild toxicities demonstrate a different priority of correlations. When limiting the RF model to eyes that do not include severe disease, ORT dominates as the most important feature in predicting function revealing the importance of ORT in the early subtle changes associated with early toxicity functional changes.

We observed improved predictability of visual function when the OCT based structural metrics were used in combination as part of the RF model. In general, intensity measurements (minimum intensity and mean intensity) had poor univariate linear correlations with visual field sensitivity but still effectively contributed to the RF model. Identification of additional independent OCT-based features and measures may have the ability to improve these predictions further.

Objective evaluation of these structural OCT features, in addition to providing the basis for RF-based prediction of visual field sensitivity, confirms their importance in revealing pathologic structural changes. Clinicians have used EZ loss as a marker of significant photoreceptor loss and evidence of severe toxicity and this model reassuringly confirms the importance of these OCT features in the RF predictive model. The model allows us to further understand the impact of these features in not only identifying changes of toxicity, but also allowing quantification of its contribution to retinal sensitivity. In a cohort of eyes without significant EZ loss, the model identifies a different OCT measure, ORT, as being the key structural measure that is the most predictive of retinal sensitivity, and solidifies its structural importance, especially in the mildly affected eyes.

Investigations into retina structure and visual function have been previously made in the fields of glaucoma and age-related macular degeneration (AMD). Histopathologic studies by Quigley et al. have reported variable amounts of retinal ganglion cell loss associated with visual field loss.^22^ Studies generating functional predictions in glaucoma have probed the relationship between OCT based measures and perimetry derived sensitivities with results ranging from MAE of 2.5 dB to 9.5 dB.^23^^–^^26^ Correlations between OCT structure and visual field sensitivities had been done in retinitis pigmentosa (RP) finding that outer retina measures were correlated with field sensitivity.^27^ In neovascular age-related macular degeneration (nAMD), OCT-based retina structure has been used to develop models predicting microperimetry sensitivities.^19^ In this study, we expand the interrogation of structure and function using nine OCT measures in the setting of an outer retinal degeneration stemming from HCQ toxicity. Our algorithm's ability to predict visual field sensitivity using nine OCT metrics is evidenced by a performance with a RMSE of 3.8 dB and MAE of 2.5 dB.

The different accuracies reported in these various models predicting functional sensitivity could depend on a multitude of factors such as the underlying disease, the fraction of the population showing disease-induced sensitivity changes, and/or the quality of the predictors. Additionally, the choice of functional test, and the limit of having a single functional test performed could limit predictability. Utilization of functional tests with higher spatial resolutions, such as those performed by Fink et al.,^28^ the potential use of a 3D-computer threshold Amsler grid test,^29^^,^^30^ or repeated measures of visual function, could potentially lead to more accurate modeling and could be interesting to include in future work.

In addition to confirming the relevant structural metrics that are clinically important, the model's approach is helpful to understand the functional consequences to retinal changes. Corroborating functional and structural findings underly the AAO recommendations for annual screening for HCQ toxicity to include visual fields and, if not reliable, mfERG testing, obtained together with structural assessments using SD-OCT.

Limitations of this study include the clinical study population, who may have been better performers on the visual field than patients screened outside the setting of clinical studies. A further limitation is the exposure of the control group to long-term HCQ. Although this group did not show any evidence of abnormality on any test modality, they may have differences from populations not taking HCQ. The small population of patients constrained the machine learning approaches to an LOPO approach. Future studies will ideally focus on obtaining a large sample size, which would allow for the implementation of more aggressive validation methods.

The results of these structure-function analyses and methods used here will be relevant to analyses in a variety of retinopathies. Other conditions causing outer retinal degenerations can also be investigated using similar methods, including retinitis pigmentosa. The application of this model to other diseases leading to outer retinal degenerations could identify similarities and differences in the structure-function relationships across disease etiologies. This in turn can lead to information regarding disease pathogenesis and outcome measures. As this technology develops, it has potential to both add to the current tools used for HCQ toxicity detection and yield insights to structure-function relationships in the retina.

## Supplementary Material

Supplement 1

Supplement 2

Supplement 3

## References

[1] Marmor MF, Kellner U, Lai TYY, Melles RB, Mieler WF, Lum F. Recommendations on Screening for Chloroquine and Hydroxychloroquine Retinopathy (2016 Revision). *Ophthalmology*. 2016; 123(6): 1386–1394. 26992838 10.1016/j.ophtha.2016.01.058

[2] Lally DR, Heier JS, Baumal C, et al. Expanded spectral domain-OCT findings in the early detection of hydroxychloroquine retinopathy and changes following drug cessation. *Int J Retin Vitreous*. 2016; 2: 18. 10.1186/s40942-016-0042-y PMC5088472 27847636

[3] Chen E, Brown DM, Benz MS, et al. Spectral domain optical coherence tomography as an effective screening test for hydroxychloroquine retinopathy (the “flying saucer” sign). *Clin Ophthalmol*. 2010; 4(1): 1151–1158. 21060664 10.2147/OPTH.S14257 PMC2964950

[4] Garrity ST, Jung JY, Zambrowski O, et al. Early hydroxychloroquine retinopathy: Optical coherence tomography abnormalities preceding Humphrey visual field defects. *Br J Ophthalmol*. 2019; 103(11): 1600–1604. 30819690 10.1136/bjophthalmol-2018-313350

[5] Marmor MF, Melles RB. Disparity between visual fields and optical coherence tomography in hydroxychloroquine retinopathy. *Ophthalmology*. 2014; 121(6): 1257–1262. 24439759 10.1016/j.ophtha.2013.12.002

[6] Allahdina AM, Stetson PF, Vitale S, et al. Optical coherence tomography minimum intensity as an objective measure for the detection of hydroxychloroquine toxicity. *Investig Ophthalmol Vis Sci*. 2018; 59(5): 1953–1963. 29677357 10.1167/iovs.17-22668 PMC5894928

[7] De Sisternes L, Hu J, Rubin DL, Marmor MF. Analysis of inner and outer retinal thickness in patients using hydroxychloroquine prior to development of retinopathy. *JAMA Ophthalmol*. 2016; 134(5): 511–519. 26986043 10.1001/jamaophthalmol.2016.0155

[8] Cukras C, Huynh N, Vitale S, Wong WT, Ferris FL, Sieving PA. Subjective and objective screening tests for hydroxychloroquine toxicity. *Ophthalmology*. 2015; 122(2): 356–366. 25444344 10.1016/j.ophtha.2014.07.056 PMC8356134

[9] Hood DC, Bach M, Brigell M, et al. ISCEV standard for clinical multifocal electroretinography (mfERG) (2011 edition). *Doc Ophthalmol*. 2012; 124(1): 1–13. 10.1007/s10633-011-9296-8 PMC4466109 22038576

[10] Tsang AC, Ahmadi S, Hamilton J, et al. The Diagnostic Utility of Multifocal Electroretinography in Detecting Chloroquine and Hydroxychloroquine Retinal Toxicity. *Am J Ophthalmol*. 2019; 206: 132–139. 31078540 10.1016/j.ajo.2019.04.025

[11] Lyons JS, Severns ML. Detection of Early Hydroxychloroquine Retinal Toxicity Enhanced by Ring Ratio Analysis of Multifocal Electroretinography. *Am J Ophthalmol*. 2007; 143(5): 801–809.e2. 17336914 10.1016/j.ajo.2006.12.042

[12] De Silva TS, Jayakar G, Grisso P, Chew EY, Hotaling N, Cukras CA. Automatic detection of ellipsoid zone loss due to Hydroxychloroquine retinal toxicity from SD-OCT imaging. In: Drukker K, Mazurowski MA, eds. *Medical Imaging 2021: Computer-Aided Diagnosis*. Vol 11597. San Diego, CA: SPIE; 2021: 23.

[13] De Silva T, Jayakar G, Grisso R, Hotaling N, Chew EY, Cukras CA. Deep-learning based automatic detection of ellipsoid zone loss in SD-OCT for hydroxychloroquine retinal toxicity screening. *Ophthalmol Sci*. 2021; 4: 100060. 10.1016/j.xops.2021.100060 PMC9560656 36246938

[14] Zhang Z, Buitendijk GH, Lee K, et al. Validity of Automated Choroidal Segmentation in SS-OCT and SD-OCT. *Invest Ophthalmol Vis Sci*. 2015; 56(5): 3202–3211. 26024104 10.1167/iovs.14-15669 PMC4451615

[15] Abramoff MD, Garvin MK, Sonka M. Retinal imaging and image analysis. *IEEE Rev Biomed Eng*. 2010; 3: 169–208. 22275207 10.1109/RBME.2010.2084567 PMC3131209

[16] Bogunovic H, Sonka M, Kwon YH, Kemp P, Abramoff MD, Wu X. Multi-surface and multi-field co-segmentation of 3-D retinal optical coherence tomography. *IEEE Trans Med Imaging*. 2014; 33(12): 2242–2253. 25020067 10.1109/TMI.2014.2336246 PMC4326334

[17] Tomasi C, Manduchi R. Bilateral filtering for gray and color images. *Sixth International Conference on Computer Vision IEEE Cat. No. 98CH36271* 1998: 839–846.

[18] Harris CR, Millman KJ, van der Walt SJ, et al. Array programming with {NumPy}. *Nature*. 2020; 585: 357–362. 32939066 10.1038/s41586-020-2649-2 PMC7759461

[19] von der Emde L, Pfau M, Dysli C, et al. Artificial intelligence for morphology-based function prediction in neovascular age-related macular degeneration. *Sci Rep*. 2019; 9(1): 11132. 31366903 10.1038/s41598-019-47565-y PMC6668439

[20] Boulesteix A-L, Janitza S, Kruppa J, König IR. Overview of random forest methodology and practical guidance with emphasis on computational biology and bioinformatics. *Wiley Interdiscip Rev Data Min Knowl Discov*. 2012; 2(6): 493–507.

[21] Hasan H, Lotery A, Price EJ, Smith GT. An objective method of diagnosing hydroxychloroquine maculopathy. *Eye (Lond)*. 2021; 35: 1922–1929. 32929180 10.1038/s41433-020-01174-6 PMC8225631

[22] Quigley HA, Dunkelberger GR, Green WR. Retinal ganglion cell atrophy correlated with automated perimetry in human eyes with glaucoma. *Am J Ophthalmol*. 1989; 107(5): 453–464. 2712129 10.1016/0002-9394(89)90488-1

[23] Hood DC, Tsamis E, Bommakanti NK, et al. Structure-function agreement is better than commonly thought in eyes with early glaucoma. *Investig Ophthalmol Vis Sci*. 2019; 60(13): 4241–4248. 31618760 10.1167/iovs.19-27920 PMC6860999

[24] Guo Z, Kwon YH, Lee K, et al. Optical coherence tomography analysis based prediction of Humphrey 24-2 visual field thresholds in patients with glaucoma. *Investig Ophthalmol Vis Sci*. 2017; 58(10): 3975–3985. 28796875 10.1167/iovs.17-21832 PMC5552000

[25] Asano S, Asaoka R, Murata H, et al. Predicting the central 10 degrees visual field in glaucoma by applying a deep learning algorithm to optical coherence tomography images. *Sci Rep*. 2021; 11(1): 2214. 33500462 10.1038/s41598-020-79494-6 PMC7838164

[26] Christopher M, Bowd C, Belghith A, et al. Deep Learning Approaches Predict Glaucomatous Visual Field Damage from OCT Optic Nerve Head En Face Images and Retinal Nerve Fiber Layer Thickness Maps. *Ophthalmology*. 2020; 127(3): 346–356. 31718841 10.1016/j.ophtha.2019.09.036 PMC8063221

[27] Rangaswamy N V., Patel HM, Locke KG, Hood DC, Birch DG. A comparison of visual field sensitivity to photoreceptor thickness in retinitis pigmentosa. *Investig Ophthalmol Vis Sci*. 2010; 51(8): 4213–4219. 20220048 10.1167/iovs.09-4945 PMC2910646

[28] Fink W, Sadun AA. Three-dimensional computer-automated threshold Amsler grid test. *J Biomed Opt*. 2004; 9(1): 149–153. 14715067 10.1117/1.1625952

[29] Kovalevskaya M, Burdenko VN. 3D-CTAG Testing of Functional and Structural Changes of the Macula. *Adv Ophthalmol Vis Syst.* 2016; 4(2): 45–50.

[30] Nazemi PP, Fink W, Sadun AA, Francis B, Minckler D. Early detection of glaucoma by means of a novel 3D computer-automated visual field test. *Br J Ophthalmol*. 2007; 91(10): 1331–1336. 17504855 10.1136/bjo.2007.116103 PMC2001017

