# Supplementary material for: Visual Field Sensitivity Prediction Using Optical Coherence Tomography Analysis in Hydroxychloroquine Toxicity
Source: Invest Ophthalmol Vis Sci. 2022 Jan 11;63(1):15. doi: 10.1167/iovs.63.1.15 (PMC8762675; doi:10.1167/iovs.63.1.15)
Supplement: Supplement 1 [file iovs-63-1-15_s001.pdf]

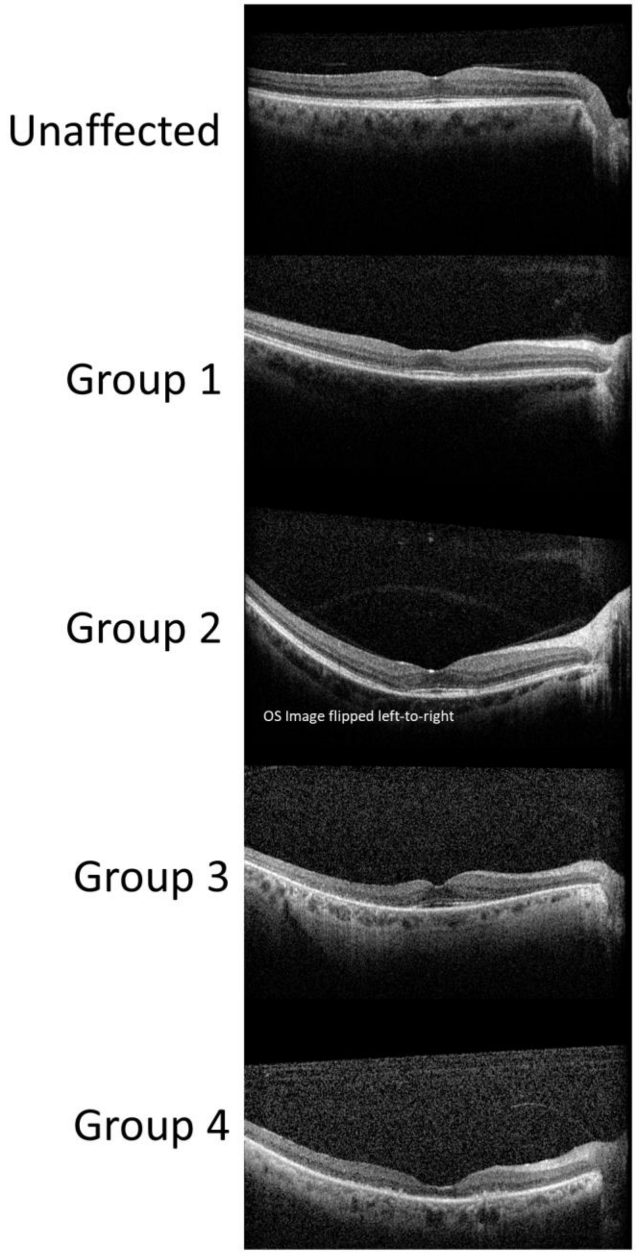

Supplementary Figure 1: Sample foveal B-scans from each of the severity groups from the Heidelberg Spectralis HRA-OCT system. Group 1: <=100 µm EZ loss on the foveal B scan, Group 2: 100 µm < EZ loss <=1000 µm on the foveal B scan, Group 3: EZ loss > 1000 µm but preserving the foveal island > 500 µm, Group 4: Foveal involvement with <= 500 involvement µm remaining in the foveal area.
