# Supplementary material for: Visual Field Sensitivity Prediction Using Optical Coherence Tomography Analysis in Hydroxychloroquine Toxicity
Source: Invest Ophthalmol Vis Sci. 2022 Jan 11;63(1):15. doi: 10.1167/iovs.63.1.15 (PMC8762675; doi:10.1167/iovs.63.1.15)
Supplement: Supplement 2 [file iovs-63-1-15_s002.pdf]

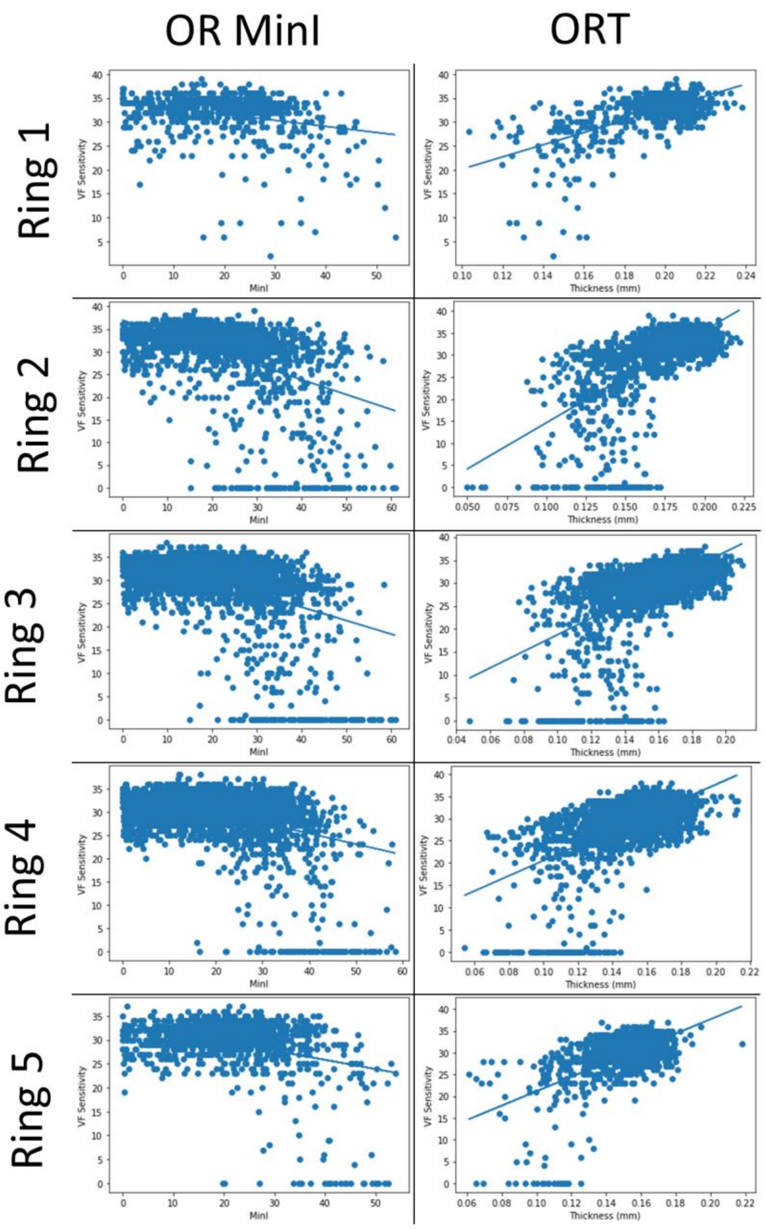

Supplementary Figure 2: Correlations between OR MinI (Left) and ORT (Right) with VF sensitivity across the different rings. Each locus in each eye is represented.
