# Supplementary material for: Visual Field Sensitivity Prediction Using Optical Coherence Tomography Analysis in Hydroxychloroquine Toxicity
Source: Invest Ophthalmol Vis Sci. 2022 Jan 11;63(1):15. doi: 10.1167/iovs.63.1.15 (PMC8762675; doi:10.1167/iovs.63.1.15)
Supplement: Supplement 3 [file iovs-63-1-15_s003.pdf]

Modality	Threshold
VF	3+ Contiguous abnormal points (p<%2) OR Full ring scotoma
SD-OCT	Evidence of photoreceptor loss and EZ disruption
mfERG	Ring 1 to ring 2 ratio greater than 2.6 OR R1 central absolute amplitude less than 35 µV

Supplementary Table 1: Thresholds for toxicity determination in subjective (perimetry) testing and objective (SD-OCT or mfERG) testing.
